# Clinical Efficacy and Safety of Photobiomodulation Therapy for Orofacial Conditions in Older Adults: A Systematic Review of Randomized Controlled Trials

**DOI:** 10.3390/dj14040231

**Published:** 2026-04-13

**Authors:** Suwat Tanya, Patcharawan Srisilapanan

**Affiliations:** 1 Department of Advanced General Dentistry and Dental Public Health, Faculty of Dentistry, Chiang Mai University, Chiang Mai 50200, Thailand; 2 School of Dentistry, University of Phayao, Phayao 56000, Thailand

**Keywords:** photobiomodulation therapy, older adults, geriatric dentistry, oral conditions

## Abstract

**Background/Objectives**: Photobiomodulation therapy (PBMT) is a non-invasive therapeutic modality that enhances tissue healing, modulates inflammation, and reduces pain. Despite increasing clinical use, evidence regarding PBMT in geriatric oral conditions has not been comprehensively synthesized. This systematic review aimed to evaluate the clinical efficacy and safety of PBMT in managing orofacial conditions in older adults. **Methods:** A systematic search of PubMed, Embase, Scopus, and Google Scholar was conducted to identify randomized controlled trials (RCTs) published between January 2000 and March 2025. Eligible studies included patients aged ≥60 years receiving PBMT for orofacial conditions. Study selection followed predefined criteria. Risk of bias was assessed using the Cochrane Risk of Bias 2 tool, and findings were narratively synthesized. **Results:** Twenty-three RCTs were included. Evidence for PBMT was most frequently reported in cancer therapy-induced oral mucositis (n = 8), with consistent reductions in lesion severity and pain. Studies on burning mouth syndrome (n = 7) and hyposalivation (n = 2) generally reported improvements in symptoms, although placebo effects were noted. Fewer studies evaluated postoperative pain (n = 2), oral lichen planus (n = 1), peri-implant conditions (n = 1), and implant osseointegration (n = 2). No clinically significant adverse events were reported. However, heterogeneity in PBMT parameters and outcome measures limited comparability. **Conclusions:** PBMT is a safe and clinically effective adjunctive therapy for managing orofacial conditions in older adults, particularly oral mucositis. These findings support its integration into geriatric oral care. Standardized protocols and well-designed RCTs are needed to determine optimal treatment parameters and long-term effectiveness.

## 1. Introduction

Aging is increasingly recognized as a nonlinear biological process characterized by dynamic molecular and physiological transitions occurring across the human lifespan. Recent multi-omics investigations have identified distinct waves of molecular dysregulation around 44 and 60 years of age, reflecting systemic alterations in immune regulation, metabolism, and tissue homeostasis that contribute to the increased risk of age-related diseases and functional decline [1]. In the oral cavity, aging is associated with structural and functional changes in the oral mucosa, including epithelial thinning, reduced keratinocyte proliferation, flattening of rete ridges, decreased fibroblast activity, diminished collagen synthesis, and reduced vascularization. These alterations increased tissue fragility, impaired regenerative capacity, and delayed wound healing [2]. Consequently, older patients were more susceptible to several orofacial conditions and complications. Clinically, aged oral mucosa was more vulnerable to cancer therapy-induced oral complications, including oral mucositis (OM), ulceration, and mucosal pain [3]. Among patients receiving radiotherapy or chemoradiotherapy, the prevalence of OM has been reported to range from approximately 38% to 80%, depending on treatment protocols and patient-related factors [4,5]. Age-related epithelial atrophy, neurosensory alterations, and salivary gland hypofunction have also been associated with burning mouth syndrome (BMS) and persistent oral burning sensations. Additionally, reduced salivary gland function contributed to hyposalivation and xerostomia, which compromised mucosal lubrication and delayed tissue repair [6]. Approximately 3% of female older than 50-year-olds was affected by BMS [7], whereas xerostomia was reported in up to 30% of older individuals [8]. Aging may further exacerbate chronic inflammatory conditions such as oral lichen planus (OLP) [9], with a prevalence of approximately 2% in people aged 40 and older adults [10]. Aging may further negatively influence implant osseointegration by reducing osteoblastic activity and slowing bone remodeling [11]. Given these biological changes, therapeutic strategies that enhanced tissue repair and modulated inflammatory responses are particularly important in older patients.

Photobiomodulation therapy (PBMT), first introduced by Endre Mester in 1967, has emerged as a promising therapeutic modality for modulating biological processes and promoting tissue repair [12]. Over the past two decades, clinical research investigating PBMT has expanded substantially across both medical and dental disciplines. From 2000 to 2025, the number of randomized controlled trials (RCTs) evaluating the clinical efficacy of PBMT increased markedly, with more than 50 PBMT-related RCT published annually in the PubMed database [13]. Accumulating evidence indicates that PBMT effectively enhanced wound healing, reduced inflammation, and alleviated pain through photochemical and photobiomodulation mechanisms [14,15].

In dentistry, recent systematic reviews have demonstrated the therapeutic potential of PBMT across a wide range of clinical applications. In terms of tissue repair, PBMT has been shown to accelerate healing of oral wounds [16,17,18], oral aphthous ulcers [19] and oral lichen planus [20]. PBMT has also demonstrated efficacy in enhancing salivary flow in patients with hyposalivation [21] and in the management of maxillofacial neuropathies [22]. Furthermore, PBMT has been reported to reduce the severity of oral mucositis [23] and alleviate symptoms of burning mouth syndrome [24]. In addition, evidence supports the adjunctive use of PBMT in the management of periodontal diseases [17,25], peri-implantitis [18,26,27], temporomandibular disorders [28,29] and root canal disinfection [30,31].

Despite the growing body of evidence supporting the clinical benefits of PBMT, its specific application in geriatric oral care remains insufficiently synthesized. Existing studies are fragmented across diverse clinical conditions, with substantial heterogeneity in PBMT parameters, outcome measures, and study designs. Moreover, limited integration of biological mechanisms, laser parameters, and clinical outcomes has hindered the translation of current evidence into standardized clinical protocols for older adults. Only one systematic review has explored the use of PBMT in older adults, with limited emphasis on orofacial conditions [32]. Therefore, a comprehensive and clinically oriented synthesis of randomized controlled trials is needed to clarify the therapeutic role and optimize the clinical application of PBMT in geriatric dentistry. This systematic review aimed to evaluate the existing RCTs assessing the clinical efficacy and safety of PBMT when applied to the oral and maxillofacial region in older adults aged 60 years and above.

## 2. Materials and Methods

This systematic review was designed in accordance with the Preferred Reporting Items for Systematic Reviews and Meta-Analyses (PRISMA) guideline 2020 [33]. The PRISMA 2020 checklist was provided as Supplementary File S1. In addition, this review protocol was also registered with PROSPERO under the number CRD420251012968.

### 2.1. Focused Question

In geriatric patients (≥60 years) with orofacial conditions, what is the clinical efficacy and safety of photobiomodulation therapy compared with sham, no intervention, or conventional treatment?

### 2.2. Study Selection Criteria

The eligibility criteria for this systematic review were established prior to the literature search, following the PICOS framework (Population, Intervention, Comparison, Outcome and Study design). Studies were included if they involved a population with a mean or median age of ≥60 years, representing older adults. Studies including mixed-age populations were eligible only if data for older participants could be clearly identified or if the overall mean age met the inclusion criterion. The intervention of interest was PBMT applied to the oral and maxillofacial region using any wavelength, power output, or irradiation protocol. Eligible comparison groups included those receiving no PBMT, sham, placebo lasers, or conventional therapies (e.g., pharmacological or standard dental care). Studies were required to report clinical outcomes such as wound healing, pain reduction, bleeding control, inflammation, salivary flow, oral function, overall recovery, oral indices or patient-reported outcomes. Only RCTs were considered for inclusion. Exclusion criteria for this systematic review encompassed studies involving participants with a mean or median age of <60 years or lacking clearly identifiable data for older adults. Studies employing high-intensity or ablative laser therapies were excluded to ensure consistency with PBMT. Non-randomized designs, including observational studies, case reports, case series, reviews, editorials, and conference abstracts, were not considered. Studies that did not report clinically relevant orofacial outcomes, were unavailable in full text, or were published in languages other than English were also excluded.

### 2.3. Search Strategy

In April 2025, a comprehensive search was conducted in the PubMed, Embase, Scopus, and Google Scholar electronic databases to identify RCTs that met the eligibility criteria. The search strategy focused on titles and abstracts, using specific keywords to retrieve studies investigating the use of PBMT in older populations. After eliminating duplicates, the two researchers independently screened the titles and abstracts of all retrieved articles. Any disagreements regarding study inclusion were resolved through discussion until a consensus was reached. The following combination of terms and Boolean operators was used to conduct literature searches for studies published from January 2000 to March 2025: (“Aged” OR “older adults” OR elderly OR senior OR geriatrics OR “mean age 60” OR “≥60 years”) AND (“photobiomodulation therapy” OR “PBMT” OR “low-level laser therapy” OR “LLLT” OR “low-intensity laser therapy” OR “LILT” OR “laser therapy” OR “light therapy”) AND (“orofacial” OR “oral cavity” OR “mouth” OR “jaw” OR “maxillofacial” OR “dentistry”) AND (“clinical trial” OR “randomized controlled trial” OR “RCT”). The detailed search strategies were provided in Appendix A (Table A1 and Table A2). Publications such as reviews, case reports, editorials and correspondence were excluded. Additionally, the reference lists of all included articles were manually reviewed to identify any further relevant studies. Deduplication of the articles was performed by Rayyan’s automated deduplication function, followed by manual verification to ensure accuracy. The selection process strictly followed the predefined eligibility criteria.

### 2.4. Risk of Bias Assessment and Data Extraction

Two reviewers (S.T. and P.S.) independently screened all records, assessed study eligibility, and extracted data using a standardized data extraction form. Any discrepancies were resolved through discussion until consensus was reached. Risk of bias was independently assessed by both reviewers using the Cochrane Risk of Bias 2 (RoB 2) tool [29], which evaluated five domains: (1) bias arising from the randomization process, (2) bias due to deviations from intended interventions, (3) bias due to missing outcome data, (4) bias in measurement of the outcome, and (5) bias in selection of the reported result. Each study was classified as having low risk, some concerns, or high risk of bias according to the RoB 2 guidelines.

The extracted data included the first author, year of publication, study design, article title and clinical outcomes. The RCTs that met the inclusion criteria were independently extracted by two researchers (S.T. and P.S.) using the same data extraction form. The following data were extracted: study characteristics (the first author, year of publication, study location, RCT design and duration, sample size, sex distribution, intervention and control groups, and the average age of participants), PBMT parameters (the laser device used, wavelength, power output, irradiation mode, irradiation time, energy delivered, spot size, number of treatment sessions, and irradiation sites) and results of the studies (outcome measurements, significant findings, reported clinical efficacy, and any adverse effects associated with PBMT). The data extraction process was not formally pilot-tested. Differences in data extraction were resolved through discussion until the agreement was obtained.

Due to substantial heterogeneity in study designs, PBMT parameters, and outcome measures, a meta-analysis was not performed, and findings were synthesized using a structured narrative approach. Consequently, formal assessment of publication bias was not conducted. However, the included studies reported both statistically significant and non-significant findings, suggesting that the synthesis was not limited to studies with positive outcomes.

## 3. Results

### 3.1. Search Results and Study Selection

**Figure 1:**
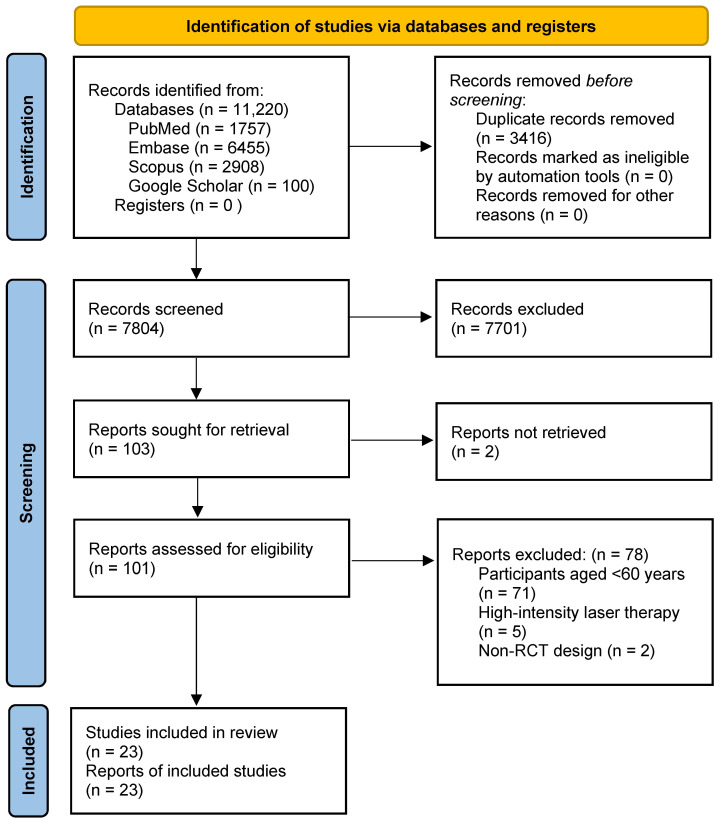
Identification of studies based on PRISMA 2020 [33].

The database search identified a total of 11,220 records across PubMed (n = 1757), Embase (n = 6455), Scopus (n = 2908), and Google Scholar (n = 100). Due to the large volume of results and the decreasing relevance of records beyond the initial pages, only the first 100 records from Google Scholar were screened, consistent with common practice in systematic reviews. After removal of duplicates, 7804 records remained for title and abstract screening, of which 7701 were excluded based on predefined eligibility criteria. A total of 101 full-text articles were assessed for eligibility, and 78 studies were excluded due to ineligible population (n = 71), use of high-intensity laser therapy (n = 5), non-randomized design (n = 2), or unavailable full text (n = 2). Ultimately, 23 randomized controlled trials met the inclusion criteria and were included in the final analysis. (Figure 1). This rigorous selection process highlights the limited availability of high-quality RCTs specifically investigating PBMT in geriatric populations.

The 23 included RCTs were conducted across 11 countries, reflecting broad geographical representation and increasing global interest in PBMT applications in geriatric dentistry. Sample sizes ranged from 12 to 78 participants, with all studies including individuals aged 60 years or older. The included studies evaluated a wide range of clinical applications, including prevention and management of cancer therapy-induced oral complications, burning mouth syndrome, postoperative pain, hyposalivation, oral lichen planus, peri-implantitis, and implant osseointegration. Most trials employed single-, double-, or triple-blinded randomized designs, with intervention durations ranging from several days to 12 months of follow-up. Detailed characteristics of the included studies are presented in Table 1. However, substantial heterogeneity was observed across studies in terms of PBMT parameters, treatment protocols, and outcome measures, which limited direct comparability and precluded quantitative meta-analysis.

### 3.2. Analysis of Risk of Bias

**Figure 2:**
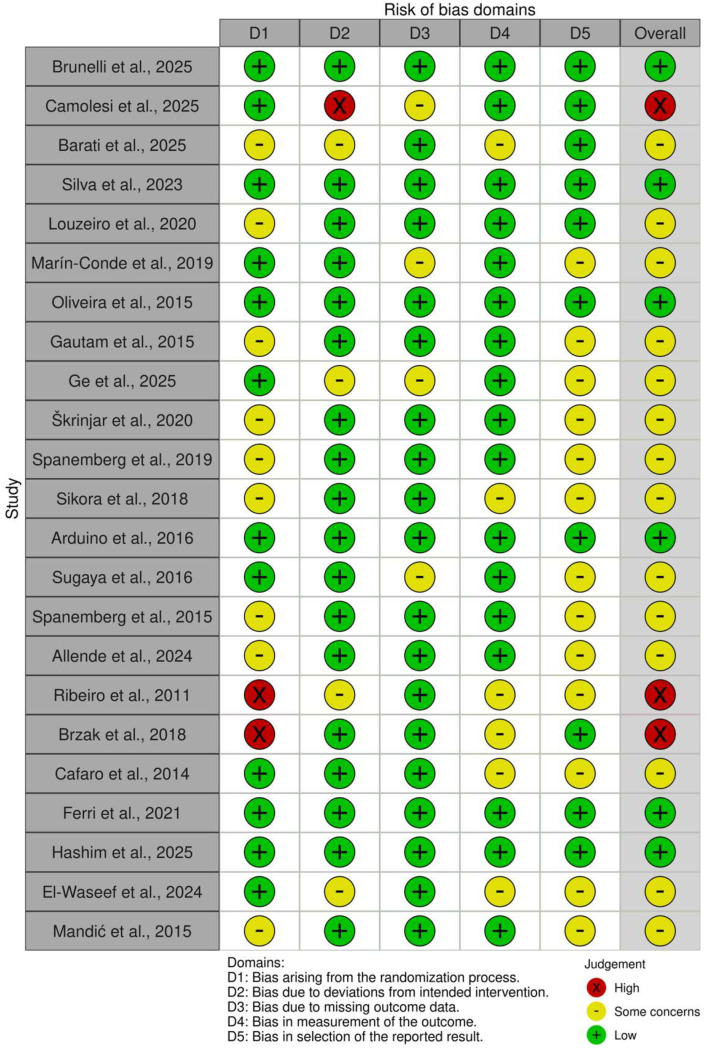
Risk of bias assessment of included randomized controlled trials using the Cochrane Risk of Bias 2 tool [34,35,36,37,38,39,40,41,42,43,44,45,46,47,48,49,50,51,52,53,54,55,56].

**Figure 3:**
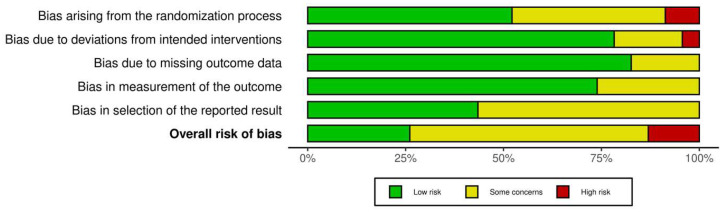
Summary of risk-of-bias judgments across domains for included randomized controlled trials assessed using the Cochrane RoB 2 tool.

Risk of bias was assessed using RoB 2 tool across five domains: randomization process, deviations from intended interventions, missing outcome data, outcome measurement, and selection of reported results. Six trials were judged to have an overall low risk of bias, demonstrating adequate randomization procedures, appropriate blinding, minimal missing outcome data, and clearly prespecified outcome analyses. The majority of studies were assessed as having some concerns, primarily due to incomplete reporting of allocation concealment and the absence of predefined statistical analysis plans. Three trials were classified as having a high risk of bias, mainly related to insufficient reporting of randomization procedures, lack of blinding, or potential selective outcome reporting. The detailed domain-level assessments are presented in Figure 2 and Figure 3. Overall, the methodological quality of the included studies can be considered moderate, with limitations primarily related to reporting transparency rather than fundamental study design. These factors should be considered when interpreting the clinical findings of this review.

### 3.3. PBMT’s Clinical Efficacy and Safety in Geriatric Dentistry

Across the included randomized controlled trials, the strength of evidence varied according to clinical application. The strength of evidence was interpreted based on the consistency of findings across studies and the potential influence of placebo effects. Conditions with consistent positive outcomes and no evident placebo effects were considered to have stronger supporting evidence, whereas conditions with a limited number of studies or with potential placebo influence were interpreted more cautiously as having moderate or limited evidence. The clinical outcomes and reported adverse events of the included studies were summarized in Table 2.

The strongest and most consistent evidence was observed for the prevention and management of cancer therapy-induced oral mucositis, with eight RCTs demonstrating consistent improvements in clinical and patient-reported outcomes. Most studies consistently reported reductions in oral mucositis severity, delayed lesion onset, and decreased pain intensity in patients receiving PBMT during radiotherapy or chemoradiotherapy [35,36,39]. Improvements in patient-reported quality of life and reductions in analgesic consumption were also observed [36]. In addition, several studies reported improvements in patient-reported quality of life and reduced analgesic consumption, supporting the clinical relevance of PBMT in this setting.

Moderate evidence was identified for the management of burning mouth syndrome (n = 7) and hyposalivation (n = 2). RCTs evaluating PBMT for burning mouth syndrome reported reductions in burning pain intensity and improvements in oral health-related quality of life following treatment [42,43,44,45,46,47,48,49]. The wavelengths used in these studies ranged from 685 to 830 nm and were delivered over multiple treatment sessions. In some trials, improvements were observed in both PBMT and sham groups [45]. Studies evaluating salivary gland irradiation reported increases in salivary flow following PBMT treatment compared with baseline or control conditions [51,52].

In contrast, evidence for postoperative pain (n = 2), oral lichen planus (n = 1), peri-implant conditions (n = 1), and implant osseointegration (n = 2) remained limited. Although some studies reported reductions in postoperative pain, improvements in inflammatory parameters, and enhanced implant stability, findings were inconsistent. RCTS investigating postoperative pain reported lower pain scores in the PBMT group compared with control conditions following oral surgical procedures [50]. Additional studies evaluated the adjunctive use of PBMT in inflammatory oral diseases and peri-implant conditions. In patients with oral lichen planus, PBMT produced clinical improvements in pain and lesion severity comparable to those achieved with topical corticosteroid therapy [53]. Studies evaluating peri-implant conditions reported reductions in inflammatory parameters following adjunctive PBMT compared with mechanical debridement alone [54]. Two randomized controlled trials evaluated the effect of PBMT on implant stability and osseointegration. These studies reported higher implant stability quotient (ISQ) values and improved early implant stability in PBMT-treated implants compared with control groups [55,56].

Importantly, PBMT demonstrated an excellent safety profile across all included studies. No clinically significant adverse events related to laser irradiation were reported. Minor transient sensations, such as mild warmth during irradiation, were occasionally described but did not require intervention. Overall, PBMT appears to be a safe and clinically effective adjunctive therapy in geriatric dentistry, with the most robust evidence supporting its use in cancer therapy-induced oral mucositis.

### 3.4. Laser Parameters and Irradiation Protocols Used in the Included Studies

Considerable variability was observed in PBMT parameters across the included RCTs, reflecting differences in clinical indications and treatment objectives.

Wavelengths ranged from 632.8 to 980 nm, with red light (approximately 630 to 660 nm) and near-infrared wavelengths (approximately 808 to 830 nm) being the most frequently used. A consistent pattern emerged in which red wavelengths were predominantly applied for superficial mucosal conditions, whereas near-infrared wavelengths were more commonly used for deeper tissue targets, including salivary glands and peri-implant tissues. Of the 23 RCTs, 15 used single-wavelength PBMT, whereas 8 applied dual-wavelength PBMT regimens. Dual-wavelength protocols, typically combining intraoral red light with extraoral near-infrared irradiation, were frequently employed in the management of cancer therapy-related oral complications. Detailed PBMT parameters were summarized in Table 3.

Laser output power ranged from 5 to 500 mW, with 100 mW being the most commonly applied setting. Lower power levels were generally used in mucosal conditions while moderate power settings were more frequently applied in oral mucositis and peri-implant protocols.

Energy density varied widely across studies; however, most trials delivered between 1 and 10 J/cm^2^ per irradiation site or applying 1 to 6 J/point. This range appears to represent a clinically effective therapeutic window, consistent with the biphasic dose–response hypotheses described in PBMT. Lower energy doses were typically used in studies targeting oral mucositis and mucosal lesions, whereas higher energy densities were occasionally applied in protocols involving burning mouth syndrome or larger irradiation areas.

Irradiation time per point ranged from 5 to 381 s, with most studies applying 10 to 50 s per point, depending on the selected power output. Treatment frequency varied from single applications to repeated sessions over several weeks, with more intensive protocols commonly used in cancer therapy-related conditions. Overall, PBMT protocols were adapted according to tissue depth, clinical indication, and therapeutic goals.

Table 4 presented clinically relevant PBMT parameters derived from RCTs demonstrating superior or comparable outcomes, highlighting indication-specific protocols and a consistent therapeutic range across geriatric oral conditions.

## 4. Discussion

This systematic review synthesized evidence from RCTs evaluating the clinical efficacy and safety of PBMT in geriatric dentistry. The findings demonstrated that PBMT was a safe and clinically effective therapy for managing several orofacial conditions in older adults, with the strongest evidence observed in the prevention and management of cancer therapy-induced oral mucositis.

The therapeutic effects of PBMT were explained by its underlying photobiological mechanisms. At the cellular level, PBMT enhanced mitochondrial activity, stimulates cytochrome c oxidase, and increases adenosine triphosphate (ATP) production, while modulating reactive oxygen species and promoting nitric oxide release. These processes improved cellular metabolism, enhanced microcirculation, promoted angiogenesis, and accelerated tissue repair. In addition, PBMT modulated inflammatory pathways and reduced nociceptive signaling, contributing to its analgesic and anti-inflammatory effects [14,15]. These mechanisms were particularly relevant in older adults, in whom aging was associated with reduced cellular proliferation, impaired mitochondrial function, diminished collagen synthesis, and decreased vascularization of oral tissues, resulting in delayed wound healing and increased susceptibility to inflammatory oral diseases [1,2]. The ability of PBMT to enhance microcirculation, stimulate cellular activity, and modulate inflammation directly addressed these age-related biological limitations, supporting its clinical application in geriatric populations [57,58].

The present findings indicated that PBMT was most effective in the management of cancer therapy-induced oral mucositis. Multiple RCTs demonstrated significant reductions in mucositis severity, delayed onset of ulcerative lesions, and decreased pain intensity in patients receiving PBMT during radiotherapy or chemoradiotherapy [35,36,37,38,40,41,42]. Improvements in patient-reported outcomes, including reduced weight loss and improved quality of life, were also observed. These findings were consistent with previous systematic reviews and clinical guidelines recommending PBMT for oral mucositis management in oncology patients [3,59].

Moderate evidence was identified for BMS, a chronic neuropathic condition commonly affecting older adults. Several randomized controlled trials demonstrated reductions in burning pain intensity following PBMT treatment [44,46,47,48]. However, comparable improvements observed in sham-treated groups suggest that placebo effects and central pain modulation may contribute to clinical outcomes [43,45]. Given the multifactorial and neuropathic nature of BMS, PBMT may function primarily as a neuromodulatory therapy rather than a definitive disease-modifying intervention [60]. Mechanistically, PBMT may influence nociceptive pathways through modulation of neural activity, reduction in neuroinflammation, and enhancement of mitochondrial function in neural tissues, contributing to symptom relief [61].

PBMT also demonstrated beneficial effects in hyposalivation and xerostomia, conditions commonly associated with aging, polypharmacy, and systemic disease. Several RCTs reported significant improvements in salivary flow following PBMT application to major salivary glands [51,52]. These effects may be explained by enhanced microcirculation, stimulation of mitochondrial metabolism, and increased glandular cellular activity, resulting in improved secretory function [62].

In inflammatory oral diseases such as OLP, PBMT demonstrated reductions in pain intensity and lesion severity comparable to topical corticosteroid therapy [53]. These findings suggested that PBMT may serve as a viable alternative or adjunctive treatment, particularly in patients with contraindications to long-term corticosteroid use. The therapeutic effects were likely mediated through modulation of cytokine expression, reduction in oxidative stress, and promotion of tissue repair [63].

In peri-implant conditions and implant healing, PBMT was associated with improvements in peri-implant soft tissue health, reductions in inflammatory parameters, and enhanced early implant stability [54,55,56]. However, evidence regarding long-term implant success remained limited. Notably, surgical approaches demonstrated superior outcomes in peri-implantitis management compared with PBMT alone, although PBMT offers a minimally invasive adjunctive option [54].

An important methodological consideration identified in this review was the heterogeneity of PBMT irradiation parameters. The included studies applied wavelengths ranging from approximately 630 to 980 nm, with considerable variation in power output, energy density, irradiation time, and treatment frequency. These parameters critically determined tissue penetration and biological response, with red wavelengths primarily affecting superficial tissues and near-infrared wavelengths penetrating deeper structures [14].

Despite this variability, several patterns emerged. Effective protocols for oral mucositis commonly utilized red (approximately 630 to 660 nm) and near-infrared (approximately 800–830 nm) wavelengths delivered repeatedly during cancer therapy. In contrast, management of BMS and salivary gland dysfunction generally involved multiple sessions using near-infrared wavelengths, reflecting the need for deeper tissue penetration and neuromodulatory effects. For inflammatory conditions such as OLP, lower energy densities and localized application were frequently applied. These findings highlighted the importance of tailoring PBMT protocols according to tissue depth, disease pathophysiology, and therapeutic targets.

PBMT also exhibited a biphasic dose–response relationship, in which insufficient energy produced minimal therapeutic effects, whereas excessive energy may inhibit cellular activity [15]. However, variability in outcome measures and follow-up durations limits comparability across studies and affects interpretation of clinical effectiveness. In addition, methodological concerns, particularly related to allocation concealment and reporting transparency, were identified in some studies [29].

From a clinical perspective, PBMT represents a non-invasive, safe, and well-tolerated therapeutic modality that can be integrated into routine geriatric dental care. However, several barriers may limit its widespread implementation, including variability in laser systems, lack of standardized treatment protocols, operator training requirements, and economic considerations. Addressing these challenges through standardized clinical guidelines and training programs will be essential for broader clinical adoption.

Future research should prioritize the development of standardized PBMT protocols tailored to specific geriatric oral conditions. Large-scale, RCTs with extended follow-up periods are required to establish optimal treatment parameters and confirm long-term clinical effectiveness. Further investigation into the molecular mechanisms of PBMT in aging tissues may also enhance therapeutic precision and improve clinical outcomes.

Overall, this review provides a comprehensive synthesis of current evidence and highlights PBMT as a valuable adjunctive therapy in geriatric dentistry, particularly for conditions characterized by impaired healing and inflammation.

## 5. Conclusions

PBMT is a safe and clinically effective adjunctive modality for managing orofacial conditions in older adults, with the strongest evidence supporting its use in the prevention and management of cancer therapy-induced oral mucositis. Moderate evidence exists for its application in burning mouth syndrome and hyposalivation, while evidence for postoperative pain, oral lichen planus, and peri-implant conditions remains limited.

From a clinical perspective, PBMT may be considered as an adjunctive option for managing oral mucositis in older patients, particularly in oncology settings, and may be selectively applied in other conditions where moderate evidence supports symptom improvement. However, its use should be guided by available evidence.

The absence of standardized PBMT protocols and the heterogeneity of current evidence highlight the need for well-designed randomized controlled trials with consistent parameters and long-term follow-up. Future research should focus on establishing optimal treatment protocols and strengthening the evidence base for broader clinical applications.

Overall, PBMT represents a non-invasive therapeutic approach that may enhance clinical outcomes and improve quality of life in geriatric dental care.

## Figures and Tables

**Table 1:** Characteristics of the included randomized controlled trials.

Clinical Applications	Study	Study Site	RCT Design and Duration	Study Size, n	Male, n	Intervention and Control (n)	Average Age, y ± SD
**Prevention and management of cancer therapy-induced oral side effects**	Brunelli et al., 2025 [34]	Brazil	Participant and assessor-blinded RCT/5-week CRT	70	55	PBMT(intraoral) + sham PBMT (extraoral) (35) PBMT (intraoral + extraoral) (35)	63.0 ± 9.4
	Camolesi et al., 2025 [35]	Spain	Single-blinded RCT/6-week CRT with FU 15 days, 2, 3, 6 months	53	36	supportive care + PBMT (27) supportive care + sham PBMT (26)	64.3 ± 9.9
	Barati et al., 2025 [36]	Iran	Single-blinded RCT/1 month	36	23	PBMT (18) Mouthwash ^a^ + sham PBMT (18)	60.8
	Silva et al., 2023 [37]	Brazil	Participant and assessor-blinded RCT/5 months	53	42	PBMT intraoral + extraoral (26) Sham PBMT (27)	60.6 ± 7.1
	Louzeiro et al., 2020 [38]	Brazil	Single-blinded RCT/6 to 7-week CRT	21	16	PBMT (11) Sham PBMT (10)	64.1 ± 8.3
	Marín-Conde et al., 2019 [39]	Spain	Double-blinded RCT/9-week CRT with 3-week FU	26	20	PBMT (11) Sham PBMT (15)	60.9 ± 9.9
	Oliveira et al., 2015 [40]	Brazil	Triple-blinded RCT/1 month	16	11	PBMT daily (8) PBMT every other day (8)	66.5 ± 13.1
	Gautam et al., 2015 [41]	India	Double-blinded RCT/6.5-week CRT	46	39	PBMT (22) Sham PBMT (24)	70.6 ± 8.1
**Management of burning mouth syndrome and burning sensation**	Ge et al., 2025 [42]	China	Single-blinded RCT/4 weeks	53	5	660 nm PBMT (10) 810 nm PBMT (26) 975 nm PBMT (17)	60 (30 to 77) ^b^
	Škrinjar et al., 2020 [43]	Croatia	Double-blinded RCT/10 days	23	3	PBMT (12) Sham PBMT (11)	62 (47 to 70) ^b^
	Spanemberg et al., 2019 [44]	Spain	Double-blinded RCT/4-week intervention with 2-month FU	21	1	PBMT (12) Sham PBMT (9)	66.5 ± 6.99
	Sikora et al., 2018 [45]	Croatia	Single-blinded RCT/2 weeks	44	1	PBMT (22) Sham PBMT (22)	67.6
	Arduino et al., 2016 [46]	Italy	RCT/5-week intervention with 12-week FU	33	8	PBMT (18) Clonazepam (15)	67.1 ± 8.6
	Sugaya et al., 2016 [47]	Brazil	Triple-blinded RCT/2-week intervention with 3-month FU	23	2	PBMT (13) Sham PBMT (10)	60.0 ± 11.2
	Spanemberg et al., 2015 [48]	Brazil	Single-blinded RCT/8 weeks	78	11	830 nm PBMT, 1×/week (20) 830 nm PBMT, 3×/week (20) 685 nm PBMT (19) Sham PBMT (19)	62.2 ± 8.0
**Management of postoperative pain**	Allende et al., 2024 [49]	Chile	Double-blinded split-mouth RCT/1 week	20	10	PBMT (20) Sham PBMT (20)	77.5
	Ribeiro et al., 2011 [50]	Brazil	Double-blinded RCT/3-day intervention + 6 to 12-month FU	18	3	PBMT (8) Sham PBMT (10)	64.7 ± 11.3
**Management of hyposalivation**	Brzak et al., 2018 [51]	Croatia	RCT/10-day intervention with 10-day FU	30	0	830 nm PBMT (15) 685 nm PBM (15)	72 (52 to 85) ^b^
	Cafaro et al., 2014 [52]	Italy	Single blinded RCT 5-week intervention + 6-month FU	26	0	PBMT (14) Sham PBMT (12)	69.3 ± 9.8
**Management of OLP**	Ferri et al., 2021 [53]	Brazil	Double-blind RCT/1-month intervention + 3 months FU	34	2	Placebo gel + PBMT (17) 0.05% clobetasol + sham PBMT (17)	62.2
**Management of peri-implantitis**	Hashim et al., 2025 [54]	Switzerland	Assessor-blinded RCT/12 months	38	20	Mechanical debridement + PBMT (19) Mechanical debridement + surgery (19)	68.8 ± 11.0
**Promoting implant osseointegration**	El-Waseef et al., 2024 [55]	Egypt	Single-blinded RCT/12 months	18	8	PBMT (9) Sham PBMT (9)	60 (50 to 65) ^b^
	Mandić et al., 2015 [56]	Serbia	Single-blinded split-mouth RCT/6 weeks	12	6	PBMT (6) Sham PBMT (6)	61.3

n: number of participants; y: years; RCT: Randomized Controlled Trial; CRT: Chemoradiotherapy; PBMT: photobiomodulation therapy; FU: Follow-up; SD: standard deviation; OLP: oral lichen planus; ^a^ mouthwash containing diphenhydramine with Almgs; ^b^ Median (range).

**Table 2:** Clinical outcomes and adverse effects of photobiomodulation therapy in included randomized controlled trials.

Author	Study Objective	Outcome Measurement	Significant Finding	Clinical Efficacy	Adverse Effects
Brunelli et al., 2025 [34]	To evaluate intraoral and extraoral PBMT for prevention and management of OM and xerostomia resulting from CRT	OHIP-14 *, VAS pain score *, XeQOLS *, WHO OM grading, salivary flow using sialometry	Combined intraoral and extraoral PBMT significantly improved OHIP-14, VAS pain score, XeQOLS, WHO OM grading, salivary flow and xerostomia compared with intraoral PBMT alone (*p* < 0.001).	Combined intraoral and extraoral PBMT was superior to intraoral PBMT for prevention and management of OM and xerostomia	None reported
Camolesi et al., 2025 [35]	To assess the effect of PBMT in prevention and treatment of oral side effects during CRT	WHO OM grading *,VAS pain score, VAS dysgeusia, analgesic use, xerostomia inventory questionnaire, global salivary tests, trismus and candidiasis	Supportive care with PBMT delayed onset and reduced severity of OM (*p* < 0.001), lower VAS pain at week 5 of CRT and 15-day FU (*p* < 0.05), improved salivary flow (*p* < 0.001), and reduced VAS dysgeusia at week 6 of CRT and 15-day FU (*p* < 0.05)	Supportive care with adjunctive PBMT was superior to supportive care alone for reducing oral side effects during CRT	None reported
Barati et al., 2025 [36]	To evaluate the effectiveness of PBMT in reducing severity, pain, and discomfort of OM induced by CRT	VAS pain score, WHO OM grading, FACT-HN, OMWQ-HN, QoL and soreness	PBMT significantly reduced pain from week 1 onward (*p* = 0.001) and improved QoL compared with the mouthwash group. OM severity decreased sharply in the PBMT group at week 1 (*p* < 0.001), and by week 4, 83% of PBMT patients had no oral soreness versus 44% in controls	PBMT demonstrates superior efficacy in reducing OM severity, alleviating pain, and improving QoL.	None reported. (no moderate and extreme soreness in PBMT group)
Silva et al., 2023 [37]	To investigate PBM effectiveness in reducing OM and xerostomia in cancer patients	XeQoL *, WHO OM grading *, DMFT, periodontal indices	PBMT significantly reduced oral mucositis severity (*p* = 0.0001), improved XeQoL scores (*p* = 0.0074), and markedly reduced xerostomia prevalence at 5 months (7.7% vs. 100% in controls).	PBMT demonstrated superior efficacy in reducing OM and xerostomia	None reported
Louzeiro et al., 2020 [38]	To determine PBMT efficacy in preventing xerostomia and preserving salivary function during RT	Salivary flow rate *, salivary composition, xerostomia questionnaire, VAS, TESS, UW-QoL	Only unstimulated saliva showed a significantly higher pH at the end of RT in the PBMT group compared with the sham group (*p* = 0.037).	PBMT appeared to have no effect on preventing xerostomia.	None reported
Marín-Conde et al., 2019 [39]	To assess the effect of PBMT in prevention and treatment of OM during CRT	Oral mucositis severity * (RTOG/EORTC scale), Duration of OM, Number of mucosal sites affected, pain and analgesic use, infectious complications, adverse events	PBMT significantly reduced the severity and duration of OM, with 73% of PBMT patients were mucositis-free at week 5 compared to 20% of controls. No PBMT patients developed grade 3 OM during weeks 7–8. OM duration was significantly shorter (4 vs. 7.6 weeks, *p* < 0.01), and fewer mucosal sites were affected.	PBMT was superior in preventing severe OM, reduced duration and complications	None reported
Oliveira et al., 2015 [40]	To evaluate whether daily vs. alternate-day PBMT is more effective in preventing OM in oral cancer patients undergoing CRT	WHO OM grading *, VAS pain score, BMI, salivary flow, SGAPP, OHIP-14, DMFT index	Daily PBMT prevented progression to severe oral mucositis with lower pain and better preserved salivary flow (*p* = 0.006), while alternate-day PBM resulted in significantly more grade 2–3 OM and higher pain scores.	Daily PBMT was more effective than alternate-day PBMT in reducing oral mucositis severity and pain, and in improving salivary flow.	None reported
Gautam et al., 2015 [41]	To investigate PBMT effectiveness in reducing OM severity in elderly cancer patients undergoing RT	Incidence * and severity of OM * (RTOG/EORTC scale), VAS pain score, weight loss, opioid analgesics use, enteral feeding, need for RT break	The PBMT group had a significantly lower incidence and shorter duration of severe oral mucositis (10.5 vs. 16.1 days, *p* = 0.016) and severe oral pain (10.0 vs. 16.5 days, *p* = 0.023) compared to placebo.	PBMT was superior in reduced OM severity/duration, reduced pain, less weight loss than sham PBMT	None reported
Ge et al., 2025 [42]	To compare the efficacy of PBMT using 660 nm, 810 nm, and 975 nm in alleviating pain in primary BMS patients	Burning pain intensity (VAS) *, numbness (VAS), taste alteration (VAS)	Burning pain intensity significantly decreased in all wavelength groups (median reduction: 23.4% at 660 nm, 40% at both 810 and 975 nm), while numbness improved significantly at 810 and 975 nm.	810 nm showed the most favorable overall results	None reported
Škrinjar et al., 2020 [43]	To determine the effect of PBMT on salivary cortisol levels and burning symptom intensity in BMS patients before and after treatments	Burning pain intensity (VAS) *, Salivary cortisol level (ELISA)	VAS scores decreasing (PBMT: median 5.5 to 4; Control: 5 to 3, *p* < 0.05) and salivary cortisol levels also significantly reduced (PBMT: 0.337 to 0.305 µg/dL; Control: 0.313 to 0.222 µg/dL, *p* < 0.05).	A reduction in symptoms and cortisol levels was observed in both the PBMT and sham PBMT, suggesting a placebo effect.	None reported.
Spanemberg et al., 2019 [44]	To evaluate the effect of PBMT in reducing burning sensation	Burning pain intensity (VAS) *, HADS	Baseline VAS: 8.9 (laser) vs. 8.3 (control). After treatment: 5.5 vs. 5.8. Two-month follow-up: 4.7 vs. 5.1. Significant improvement in the laser group at 2-month follow-up (*p* = 0.0038).	PBMT showed clinical improvement in BMS symptoms and may represent an alternative therapeutic approach	None reported.
Sikora et al., 2018 [45]	To compare the efficacy of PBMT in reducing burning sensation and improving QoL	Burning pain intensity (VAS) *, OHIP-CRO 14	Pain intensity significant decrease in both PBMT and sham PBMT (*p* < 0.005).	Both PBMT and sham PBMT reduced pain, suggesting a placebo effect.	None reported.
Arduino et al., 2016 [46]	To assess the effects and safety of PBMT compared with topical clonazepam in patients with BMS	Pain perception * (VAS, McGill Pain Questionnaire, PPI), OHIP-49, Anxiety and depression (HADS, GDS)	At 12-week follow-up, PBMT significantly reduced pain scores (VAS, *p* = 0.004; McGill, *p* = 0.002; PPI, *p* = 0.002) and improved OHIP-49, (*p* = 0.010). Clonazepam improved only some pain scores. PBMT showed superior pain reduction at 8-week FU (*p* = 0.026).	PBMT resulted in greater and longer-lasting reductions in BMS-related pain than clonazepam.	32% of clonazepam group reported dizziness, headache, fever, or appetite loss.
Sugaya et al., 2016 [47]	To assess the effectiveness of PBMT in reducing symptoms of BMS compared with placebo PBMT	Burning pain (0–10 VAS) *	The PBMT group showed significantly better outcomes, with a higher proportion of patients reporting <25% burning at 2 weeks (*p* = 0.002) and no burning at day 90 (*p* = 0.02)	Outcomes favored PBMT, although spontaneous remission was also reported	None reported.
Spanemberg et al., 2015 [48]	To evaluate the effect of PBMT in reducing burning mouth symptoms	Burning pain intensity (VAS, VNS) *, OHIP-14	Significant reduction in burning symptoms in all laser groups after treatment and maintained at 8-week follow-up (*p* < 0.001). PBMT groups showed greater improvement than control.	PBMT significantly reduced BMS symptoms and improved oral health-related quality of life.	None reported.
Allende et al., 2024 [49]	To evaluate PBMT using 660 nm and 808 nm reduces postoperative pain patients undergoing dental implant surgery	Postoperative pain intensity (NRS, VRS) *, onset and duration of first pain episode, pain evolution over 7 days	Pain intensity was significantly lower in the PBMT group at 24 h (*p* = 0.019), with no significant difference between groups at 7 days (*p* = 0.331).	Single-dose PBMT reduced early postoperative pain.	None reported
Ribeiro et al., 2011 [50]	To investigate the ability of PBMT to alleviate postoperative pain caused by cryosurgery	Postoperative pain intensity (NRS) *, Edema, Recurrence rate	Pain intensity tended to be lower in the PBMT group (*p* = 0.249). Edema showed no significant difference between groups (*p* = 0.342). No recurrence occurred during the 6–12-month follow-up, and all lesions healed with normal mucosa and no scarring or infection.	Pain intensity seems to be lower in the PBMT group.	None reported
Brzak et al., 2018 [51]	To evaluate the effects of different PBM wavelengths (830 nm vs. 685 nm) on salivation in patients with hyposalivation	Unstimulated and stimulated salivary flow rate (mL/5 min) *, durability of effect 10 days after treatment	Salivary flow increased significantly in both groups after 10 days (*p* < 0.0001) and remained improved 10 days post-treatment (685 nm: *p* = 0.0121; 830 nm: *p* = 0.0347). The 830 nm group showed greater salivary flow (*p* = 0.0019)	Both PBMT regimens were effective, with greater salivary stimulation observed at 830 nm.	None reported
Cafaro et al., 2014 [52]	To evaluate the effects and efficacy of PBMT with acupuncture on salivary flow rates in patients with Sjögren’s syndrome.	Unstimulated salivary flow rate (Schirmer’s test, mm/5 min) *, durability of effect (follow-up at 1, 3, and 6 months), safety/tolerability	PBMT with acupuncture significantly improved salivary flow rate compared with placebo at all post-treatment time points (*p* < 0.005), with increasing from 5.59 ± 4.79 mm at baseline to 29.7 ± 40.9 mm at week 5 (*p* = 0.001).	PBMT with acupuncture significantly increased and sustained salivary flow compared to placebo.	None reported
Ferri et al., 2021 [53]	To compare the efficacy of PBMT versus topical clobetasol 0.05% in symptomatic oral lichen planus patient.	Pain intensity (VAS) * Thongprasom’s clinical score, functional scores (chewing, swallowing, taste, fluid intake), clinical resolution, recurrence, adverse events.	Both groups demonstrated significant pain reduction from baseline to D14, D21, and D30, which was maintained during follow-up. Complete resolution occurred in both groups at the end of treatment	PBMT showed comparable efficacy to topical 0.05% clobetasol in patients with symptomatic oral lichen planus.	None reported.
Hashim et al., 2025 [54]	To evaluate the efficacy of non-surgical debridement with repeated PBMT compared to surgical treatment for peri-implantitis management.	Resolution of peri-implantitis (PD ≤ 4 mm, no BOP, no progressive bone loss) *, PD, recession, marginal bone levels; adverse events; patient-reported discomfort (VAS)	Both groups showed significant PD reduction and bone level improvement at 3 and 12 months. However, surgical treatment achieved significantly lower PD values (*p* = 0.01) and higher treatment success, while laser therapy failed in 23.5% of cases (*p* = 0.04).	Surgical approach demonstrated superior clinical outcomes for peri-implantitis management, although diode laser therapy provided a minimally invasive technique	Surgical group reported gastrointestinal disturbances related to ABO.
El-Waseef et al., 2024 [55]	To compare the effects of PBMT and placebo on peri-implant tissues in patients with narrow mandibular ridges	PD, plaque index, bleeding index, gingival index, radiographic VBL	Bleeding index was significantly lower in the PBMT group at 3 and 6 months (*p* = 0.006 and 0.018). Gingival index was significantly lower at 3 and 6 months (*p* = 0.002 and 0.015). Peri-implant vertical bone loss was significantly greater in the control group at 6 and 12 months (*p* = 0.015 and 0.001).	PBMT improved peri-implant soft tissue health and reduced marginal bone loss around implants supporting overdentures.	None reported.
Mandić et al., 2015 [56]	To investigate the influence of PBMT on osseointegration and early success of implants placed into low-density bone of posterior maxilla.	ISQ *, ALP activity in peri-implant crevicular fluid, Early implant success rate (Buser criteria).	Implant stability was higher in the PBMT group, reaching significance at week 5 (*p* = 0.030). ALP activity showed no significant difference between groups (*p* > 0.05).	PBMT produced slightly higher implant stability but did not significantly influence overall osseointegration or early implant success.	None reported.

* primary outcome measurement; PBMT: photobiomodulation therapy; OM: oral mucositis; CRT: chemoradiotherapy; OHIP-14:Oral Health Impact Profile-14; VAS: Visual Analog Scale; WHO OM: World Health Organization oral mucositis grading; XeQOLS: Xerostomia-Related Quality of Life Scale; FACT-HN: Functional Assessment of Cancer Therapy–Head and Neck; OMWQ-HN: Oral Mucositis Weekly Questionnaire–Head & Neck; QoL: quality of life; DMFT: Decayed, Missing, and Filled Teeth; RT: radiotherapy; TESS: Treatment-Emergent Symptom Scale; UW-QoL: University of Washington Quality of Life; RTOG/EORTC: the Radiation Therapy Oncology Group/European Organization for Research and Treatment of Cancer; SGAPP: the subjective global assessment produced by the patient; BMS: burning mouth syndrome; µg/dL: ELISA: Enzyme-Linked Immunosorbent Assay; HADS: Hospital Anxiety and Depression Scale; OHIP-CRO 14: Oral Health Impact Profile—Croatian version; OHIP-49:Oral Health Impact Profile-49; Present Pain Intensity: PPI; FU: follow-up; NRS: Numeric Rating Scale; VRS: Verbal Rating Scale; PD: probing depth; BOP: bleeding on probing; ISQ: implant stability quotient; ALP: Alkaline phosphatase; VBL: vertical bone loss; ABO: antibiotics.

**Table 3:** Photobiomodulation parameters and treatment protocols used in the included randomized controlled trials.

Author	Laser Machine	Wavelength	Power Output	Irradiation Mode	Irradiation Time	Energy Density	Spot Size	Treatment Sessions and Irradiation Sites
Brunelli et al., 2025 [34]	Therapy EC, DMC (São Carlos, Brazil)	660 nm (intraoral)	100 mW	CW	10 s/point	1 J/point, 10 J/cm^2^	0.098 cm^2^	1x/week during 5-week CRT; Intraoral (26 points): labial x4/side, lip commissure x2, buccal x3/side, lateral of tongue x3/side, FOM x4, soft palate x2; Extraoral (40 points): face x8/side, submandibular x8/side e, lips x4/side
		808 nm (extraoral)						
Camolesi et al., 2025 [35]	Laser Duo (MMOptics Ltd.a., São Carlos, Brazil)	660 nm (intraoral)	100 mW, 3.33 W/cm^2^	CW	10 s/point	1 J/point, 33.33 J/cm^2^	0.03 cm^2^	5×/week during 6-week CRT; Intraoral (at least 78 points):buccal/labial mucosa, lips, tongue, palate, retromolar, uvula); Extraoral (10 points) parotid x3/side, submandibular x2/side
		808 nm (extraoral)			20 s/point	2 J/point, 66.66 J/cm^2^		
Barati et al., 2025 [36]	THOR-LX2, Photomedicine Ltd., UK	810 nm (intraoral)	200 mW	CW	30 s/point	6 J/cm^2^	1.0 cm^2^	4 consecutive days; All visible oral mucositis lesions
Silva et al., 2023 [37]	Therapy EC (DMC, São Carlos, Brazil)	660 nm (intraoral)	100 mW	CW	10 s/point	1 J/point	0.098 cm^2^	Weekly during CRT; Intraoral (21 points): lip x3/side, tongue dorsum x1, buccal mucosa x3/sides, lateral tongue x2/sides, soft palate x2, underside tongue x2; Extraoral (18 points): parotid x6/side, submandibular glands x3/side
		808 nm (extraoral)			3 s/point	0.3 J/point		
Louzeiro et al., 2020 [38]	Photon Lase III (DMC Ltd.a, São Carlos, SP, Brazil)	660 nm (intraoral)	40 mW	CW, contact mode	7 s/point	0.28 J/point, 10 J/cm^2^	0.028 cm^2^	3×/week throughout RT; Intraoral: lip commissure x2, labial x8/side buccal x12/side, hard palate x12, soft palate x4, lateral tongue x6/side, ventral tongue x6, FOM x4, sublingual gland x2; Extraoral: parotid x6/side and submandibular glands x3/side.
		810 nm (extraoral)			17.5 s/point	0.7 J/point, 25 J/cm^2^		
Marín-Conde et al., 2019 [39]	ezlase (BIOLASE, Irvine, CA, USA)	940 nm	500 mW, 13.88 W/cm^2^	CW	6 s/point	3 J/point	0.036 cm^2^	1x/week during 9 visits of CRT and continued 3 weeks post-radiotherapy (12 sessions total); Intraoral mucosa at 74 identified sites (buccal x6/side, lips x4/side, lip commissure x2 hard palate x12, soft palate x4, lateral tongue x10/side, lingual tongue x12 FOM x4)
Oliveira et al., 2015 [40]	Therapy XT (DMC, São Carlos, Brazil)	660 ± 10 nm	100 mW	CW, light-contact	n/a	1 J/point	0.028 cm^2^	Daily group: 5×/week during RT; Alternate-day group: 3×/week with placebo 2×/week; 9 points/area: Labial and buccal mucosa x1/side, lateral tongue borders x1/side, body of tongue x1, FOM x1
Gautam et al., 2015 [41]	He-Ne laser (India)	632.8 nm	0.024 W/cm^2^	CW, non-contact	125 s/point	3 J/point	1 cm^2^	5x/week; 12 intraoral (tongue lateral and ventral aspect, labial mucosa, buccal mucosa, FOM, and palate; excluding tumor site)
Ge et al., 2025 [42]	Laser-HF (Hager & Werken, Germany)	660 nm	50 mW	CW, non-contact	30 s/point	1.5 J/cm^2^	1 cm^2^	1×/week (4 sessions total); Symptomatic oral mucosa: tongue (17 points), hard palate (6 points), buccal mucosa (12 points), and both the upper and lower lips (4 points each)
		975 nm	30 mW		33 s/point	10 J/cm^2^		
	Fotona XD-2 (Ljubljana, Slovenia)	810 nm	500 mW		6 s/point	3 J/cm^2^		
Škrinjar et al., 2020 [43]	BTL-2000 (Medical Technologies, Czech Republic)	685 nm	30 mW, 0.003 W/cm^2^	CW	381 s	2 J/cm^2^	3 cm^2^	10 sessions (1x/day for 10 consecutive days); Burning sites, up to 3 sites/patients
Spanemberg et al., 2019 [44]	Thor laser^®^ diode laser	808 nm	200 mW	CW	15 s/point	3 J/point	0.088 cm^2^	Twice weekly for 4 weeks (8 sessions); applied to symptomatic oral mucosal sites
Sikora et al., 2018 [45]	GaAlAs (BTL, Prague, Czech Republic)	830 nm	100 mW	Pulse, non-contact	300 s (800 ms on, 1 ms off)	12 J/cm^2^	1 cm^2^	10 sessions over 14 days (daily except weekends); on sites with burning symptoms in oral mucosa
Arduino et al., 2016 [46]	DM980 diode (DMT, Lissone, Italy)	980 nm	300 mW, 1 W/cm^2^	CW, non-contact	10 s/point	10 J/cm^2^	0.28 cm^2^	10 sessions (2x/week for 5 weeks); All burning mucosal sites, extending 0.5 cm beyond borders
Sugaya et al., 2016 [47]	AsGaAl, QTUM00A/QUANTUM (Brazil)	790 nm	120 mW, 4 W/cm^2^	CW, scanning mode	50 s/point	6 J/point	0.03 cm^2^	4 sessions (2/week for 2 weeks, 3-day interval); Entire area affected by burning (tongue, lips, palate, buccal, gingiva, mandibular ridge)
Spanemberg et al., 2015 [48]	Thera Lase™ (DMC Equipamentos LTDA, São Carlos, Brazil)	830 nm	100mW, 3.57 W/cm^2^	CW	50 s/point	5 J/point, 176 J/cm^2^	n/a	830 nm group: 10 sessions (1x/week for 10 weeks) and 9 sessions (3x/week for 3 weeks) 685 nm group: 9 sessions (3x/week for 3 weeks); Oral mucosa sites with burning sensation (tongue apex x3, dorsum x4, sides x10, buccal mucosa x8, labial mucosa x5, hard x8, soft palate x3, alveolar ridge x3/sextants)
		685 nm	35 mW/1.25 W/cm^2^	CW	58 s/point	2 J/point, 72 J/cm^2^	n/a	
Allende et al., 2024 [49]	THERAPY EC (DMC, Brazil)	660 ± 10 nm, 808 ± 10 nm	100 mW ± 20%	CW, contact	45 s/point	4.5 J point	n/a	1 session, immediately post-surgery: 5 sites per implant (vestibular x2, palatal x2, occlusal x1)
Ribeiro et al., 2011 [50]	Whitening Lase II (DMC LTDA, São Carlos, Brazil)	660 nm	50 mW, 1.75 W/cm	CW, contact	28 s/point	1.4 J, 49 J/cm^2^	0.029 cm^2^	3 sessions (immediately and at 48 and at 72 h post-surgery): 3 points within 1 cm^2^ around cryosurgical site
Brzak et al., 2018 [51]	BTL-2000 (Medical Technologies, Prague, Czech Republic)	685 nm	30 mW, 0.0075 W/cm	Pulse, 5.2 Hz	5,2 and 1 min	1.8 J/cm^2^	4,1.6 and 0.8 cm^2^	10 consecutive daily sessions; Bilateral parotid and submandibular (extraoral), sublingual (intraoral)
		830 nm	35 mW, 0.00875 W/cm		4:17, 1:43 and 0:51 min			
Cafaro et al., 2014 [52]	Pointer Pulse (GMT2000, Laveno Mombello, Italy)	650 nm	5 mW	CW	120 s/point	19.2 J/cm^2^	0.031 cm^2^	5 weekly sessions; Acupuncture points
Ferri et al., 2021 [53]	Laser Therapy XT, DMC Equipment, São Carlos, Brazil.	660 nm	100 mW, 0.0354 W/cm^2^	CW, contact	5 s/point	0.5 J/point	0.003 cm^2^	2×/week for 4 weeks; OLP lesions (buccal mucosa, gingiva, tongue, palate, lips, alveolar ridge, floor of mouth).
Hashim et al., 2025 [54]	Wiser diode laser^®^ (Orcos Medical AG, Küsnacht, Switzerland)	810 nm (Decontamination)	2.5 W	Pulse, 50 Hz, 10 ms	30 s/site	n/a	n/a	3 sessions: baseline (day 0), day 7, and day 14; Contaminated implant surface (submucosal); transgingival peri-implant tissues
		810 nm (PBMT)	1 W/cm^2^		60 s/site			
El-Waseef et al., 2024 [55]	BemLase (Wuhan Gigaa Optronics Technology Co., Ltd.)	980 nm	100 mW	CW	10 s/point	4 J/point	n/a	5 sessions (immediately after surgery, then 2, 4, 7, and 14 days post-surgery); Labial and lingual to implants areas
Mandić et al., 2015 [56]	Medicolaser 637 (Technoline, Belgrade, Serbia)	637 nm	40 mW	CW, non-contact	n/a	6.26 J/cm^2^	n/a	Daily for 8 days (immediately after surgery and for the following 7 days); Implant site, orthoradial to implant axis

nm; nanometer; mW: miliwatt; CW: continuous wave mode; s/point: second per point; J/point: joule per point; J/cm^2^: joule per square centimeter; cm^2^: square centimeter; FOM: floor of the mouth; W/cm^2^: watt per square centimeter; CRT: chemoradiotherapy; n/a: not available; RT: radiotherapy; ms: milisecond; Pulse: pulse mode; Hz: herzt or pulse per second; OLP: oral lichen planus; PBMT, photobiomodulation therapy.

**Table 4:** Suggested photobiomodulation parameters for geriatric oral conditions based on included randomized controlled trials that demonstrated superior or comparable clinical outcomes.

Clinical Application	Wavelengths	Power Output	Energy	Sessions	Clinical Interpretation
**Cancer therapy-induced oral side effects**	Combined intra–extraoral (660 with 808/810 nm) Red light (660, 632.8 nm) Near-infrared light (810, 940 nm)	≤500 mW (most commonly 100 to 200 mW)	1 to 3 J/point	3 to 5×/week during CRT	Superior to control in most included RCT
**Burning sensation**	Near-infrared light (808, 810, 830, 790, 980 nm) Red light (685 nm)	100 to 500 mW	3 to 6 J/point	2 to 3×/week at least 2 weeks	Beneficial in several RCT, although placebo effects were also reported
**Postoperative pain**	Combined (660 with 808 nm)	100 mW	4.5 J/point	Immediate postoperative	May reduce early postoperative pain
**Hyposalivation**	Near-infrared light (830 nm) Red light (685 nm)	30, 35 mW	2 J/cm^2^	Daily for at least 10 sessions	Improved salivary flow in the included RCT
**Oral lichen planus**	Red light (660 nm)	100 mW	0.5 J/point	2×/week at least 2 weeks	Comparable to topical 0.05% clobetasol
**Promoting implant osseointegration**	Near-infrared light (980 nm) Red light (637 nm)	≤100 mW	4 J/point	3×/week for 2 weeks	May enhance early implant stability and peri-implant tissue healing during initial osseointegration

nm: nanometer; mW: milliwatt; J/point: joule per point; J/cm^2^: joule per square centimeter; CRT: chemoradiotherapy; RCT: randomized controlled trials.

## Data Availability

The original contributions presented in this study are included in the article and Supplementary Material. Further inquiries can be directed to the corresponding authors.

## Supplementary Materials

The following supporting information can be downloaded at: https://www.mdpi.com/article/10.3390/dj14040231/s1: File S1: PRISMA 2020 checklist.

## Appendix A

**Table A1:** Search strategy: keywords and MeSH terms used for literature retrieval based on study domains.

Domain	Keywords	MeSH Term
Study domain: population with a mean or median age of at least 60 years	“older adult*”, “geriatric*”, “60 year*”, “senior”, “aging population”	“aged”, “elderly”
Study determinant: photobiomodulation	“photobiomodulation*”, “PBM*”, “low-intensity laser therapy”, “LILT”, “biostimulation”, “light therapy”, “LED”	“low-level light therapy”, “LLLT”, “laser therap*”
Study outcome: oral and maxillofacial region, clinical outcomes	“orofacial”, “stomatology”, “xerostomia”, “mucositis”, “wound”, “lesion”, “healing”, “recovery”, “pain”, “bleeding control”, “inflammation”, “recovery”, “patient-reported outcomes”	“oral”, “mouth”, “facial”, “head and neck”, “xerostomia”, “mucositis” “dentistry”

**Table A2:** Detailed search strategies and number of records retrieved from each electronic database.

Electronic Database	Syntax	Number of Records
PubMed/Medline	((“Aged”[MeSH Terms] OR “elderly”[Title/Abstract] OR “older adult*”[Title/Abstract] OR “geriatric*”[Title/Abstract] OR “60 year*”[Title/Abstract] OR “senior*”[Title/Abstract] OR “aging*”[Title/Abstract])) AND ((“Low-Level Light Therapy”[MeSH Terms] OR “Laser Therapy”[MeSH Terms] OR “photobiomodulation*”[Title/Abstract] OR “PBM”[Title/Abstract] OR “PBMT”[Title/Abstract] OR “low-intensity laser therapy”[Title/Abstract] OR “LILT”[Title/Abstract] OR “biostimulation”[Title/Abstract] OR “light therapy”[Title/Abstract] OR “LED”[Title/Abstract] OR “LLLT”[Title/Abstract])) AND ((“Mouth”[MeSH Terms] OR “Oral Cavity”[Title/Abstract] OR “Dentistry”[MeSH Terms] OR “Head and Neck Neoplasms”[MeSH Terms] OR “Xerostomia”[MeSH Terms] OR “Mucositis”[MeSH Terms] OR “orofacial”[Title/Abstract] OR “stomato*”[Title/Abstract] OR “oral wound*”[Title/Abstract] OR “oral lesion*”[Title/Abstract] OR “healing”[Title/Abstract] OR “recovery”[Title/Abstract] OR “pain”[Title/Abstract] OR “bleeding”[Title/Abstract] OR “inflammation”[Title/Abstract] OR “patient reported outcome*”[Title/Abstract])) AND (“randomized controlled trial”[Publication Type] OR randomized[Title/Abstract] OR placebo[Title/Abstract] OR “randomly assigned”[Title/Abstract]) AND (“2000/01/01”[Date-Publication]: “2025/03/31”[Date-Publication])	1757
scopus	TITLE-ABS-KEY ((“older adult*” OR geriatric* OR “60 year*” OR senior* OR aging* OR aged OR elderly) AND (“photobiomodulation*” OR PBM OR PBMT OR “low-intensity laser therapy” OR LILT OR biostimulation OR “light therapy” OR LED OR LLLT OR “laser therap*”) AND (“orofacial” OR stomato* OR “oral wound*” OR “oral lesion*” OR oral OR mouth OR facial OR “head and neck” OR xerostomia OR mucositis OR healing OR recovery OR pain OR bleeding OR inflammation OR “patient reported outcome*” OR dentistry) AND (randomized OR placebo OR “randomly assigned” OR “randomized controlled trial”)) AND (PUBYEAR > 1999 AND PUBYEAR < 2026)	6455
Embase	(‘aged’/exp OR elderly:ti,ab OR ‘older adult*’:ti,ab OR geriatric*:ti,ab OR ‘60 year*’:ti,ab OR senior*:ti,ab OR aging*:ti,ab) AND (‘photobiomodulation’/exp OR ‘low level light therapy’/exp OR ‘laser therapy’/exp OR photobiomodulation*:ti,ab OR pbm:ti,ab OR pbmt:ti,ab OR ‘low-intensity laser therapy’:ti,ab OR lilt:ti,ab OR biostimulation:ti,ab OR ‘light therapy’:ti,ab OR led:ti,ab OR lllt:ti,ab) AND (‘oral cavity’/exp OR ‘dentistry’/exp OR ‘head and neck disease’/exp OR ‘xerostomia’/exp OR ‘mucositis’/exp OR orofacial:ti,ab OR stomato*:ti,ab OR ‘oral wound*’:ti,ab OR ‘oral lesion*’:ti,ab OR healing:ti,ab OR recovery:ti,ab OR pain:ti,ab OR bleeding:ti,ab OR inflammation:ti,ab OR ‘patient reported outcome*’:ti,ab) AND (‘randomized controlled trial*’/exp OR randomized:ti,ab OR placebo:ti,ab OR ‘randomly assigned’:ti,ab) AND [2000–2025]/py	2908
Google scholar	(“older adult” OR “geriatric” OR “60 year” OR “senior” OR “aging” OR “elderly”) AND (“photobiomodulation” OR “PBM” OR “PBMT” OR “low-intensity laser therapy” OR “LILT” OR “biostimulation” OR “light therapy” OR “LED” OR “LLLT” OR “laser therapy”) AND (“orofacial” OR “stomato*” OR “oral wound” OR “oral lesion” OR “oral” OR “mouth” OR “facial” OR “head and neck” OR “xerostomia” OR “mucositis” OR “healing” OR “recovery” OR “pain” OR “bleeding” OR “inflammation” OR “patient reported outcome” OR “dentistry”) AND (“randomized” OR “placebo” OR “randomly assigned” OR “randomized controlled trial*”)	First 100 records
